# Investigating the sense of agency and its relation to subclinical traits using a novel task

**DOI:** 10.1007/s00221-022-06339-1

**Published:** 2022-04-05

**Authors:** Tegan Penton, Xingquan Wang, Caroline Catmur, Geoffrey Bird

**Affiliations:** 1 MRC Social, Genetic and Developmental Psychiatry Centre, Institute of Psychiatry, Psychology and Neuroscience, King’s College London, Denmark Hill, London, SE5 8AF UK; 2 Department of Psychology, Goldsmiths University of London, London, SE14 6NW UK; 3 Department of Psychology, Institute of Psychiatry, Psychology and Neuroscience, King’s College London, Denmark Hill, London, SE5 8AF UK; 4 Department of Management, London School of Economics and Political Science, Houghton Street, London, WC2A 2AE UK; 5 Department of Experimental Psychology, University of Oxford, Oxford, OX1 3PH UK

**Keywords:** Action–outcome contingency, Sense of agency, Metacognition of agency, Individual differences, Alexithymia

## Abstract

Tasks measuring the sense of agency often manipulate the predictability of action outcomes by introducing spatial deviation. However, the extent to which spatial predictability of an outcome influences the sense of agency when spatial deviation is controlled for remains untested. We used a novel task to investigate the effect of several factors (action–outcome contingency, spatial deviation, and spatial predictability when controlling for spatial deviation of action outcomes) on the sense of agency. We also investigated trait predictors of metacognition of agency—the degree to which participants’ confidence in their agency judgements corresponds to the accuracy of those judgements. Initial and replication samples completed contingency, deviation, and predictability versions of the task. Across samples, participants’ sense of agency was impacted by action–outcome contingency and spatial deviation of action outcomes. Manipulation of the spatial predictability of action outcomes did not reliably impact the sense of agency. Metacognition of agency was related to alexithymic traits—higher alexithymia scores were associated with reduced metacognition of agency.

**Supplementary Information:**

The online version contains supplementary material available at 10.1007/s00221-022-06339-1.

## Introduction

The sense of agency is defined as one’s awareness that one is initiating, executing and controlling one’s own actions and their consequences (Jeannerod 2003). This is a crucial component of self-awareness that determines our ability to evaluate whether we are the causal agents of our actions. The sense of agency is important for attribution of internally generated actions and their consequences to the self and, conversely, attribution of externally generated events and their consequences to another. This is not only important for processes related to action monitoring and execution, but is also vital for higher order beliefs regarding our sense of control over the world (Gallagher 2000).

Two distinct forms of the sense of agency have been investigated, an implicit and explicit form. The implicit sense of agency is typically measured using indirect markers to agency such as the perceived temporal compression between a voluntary action and its consequence relative to that of an involuntary action (intentional binding; Haggard et al. 2002). The explicit sense of agency can be investigated simply by asking participants to report how much control they had over an action and its outcome (Synofzik et al. 2008). Often, experiments investigating explicit sense of agency manipulate visual feedback associated with a participant’s action and its consequence. Participants then rate whether they felt a sense of agency on a given trial or not. For instance, in some experiments, a participant may be asked to use a joystick to move a cursor on a computer screen. In some trials, the cursor will move along the path determined by the participant’s movement of the joystick. In other trials, an angular manipulation may be applied to the cursor position so that its position deviates from the path specified by the participant’s movement. This spatial deviation reduces a participant’s sense of agency relative to trials where no manipulation is applied (for examples of studies using spatial deviation paradigms see Farrer et al. 2003; Ritterband-Rosenbaum et al. 2014). Alternatively, a participant may be asked to press a button on a keyboard. Experimenters can manipulate the probability of this button press resulting in an outcome such as the appearance of a circle on a computer screen. Crucially, experimenters can also manipulate the probability of the same outcome occurring in the absence of the participant’s button press (i.e. experimenters manipulate the probability that the event has a cause external to the participant). Higher action–outcome contingency*—*when a participant’s action is more likely to cause the outcome relative to an external cause*—*is associated with a greater sense of agency (see below for examples of studies using action–outcome contingency paradigms).

Also of theoretical interest, particularly in relation to motor control and motor learning, is the extent to which individuals have an accurate representation of their degree of control. This accuracy, known as metacognition of agency, can be measured by asking participants to report their confidence in their judgements of agency, and assessing the degree to which confidence in agency judgements tracks the accuracy of those judgements (Metcalfe and Greene 2007). Research has suggested several factors that impact the explicit sense of agency and metacognition of agency. These include the degree of (experimenter-induced) temporal delay between an action and its outcome (Sato and Yasuda 2005; Shanks et al. 1989; Neunaber and Wasserman 1986), the degree of spatial deviation between the participant’s actions and their outcomes (Farrer et al. 2003; Ritterband-Rosenbaum et al. 2014), and the degree of contingency between an action and an outcome (e.g. Sidarus et al. 2013; Shanks and Dickinson 1991). However, several features of this literature mean that further work investigating factors impacting the sense of agency and metacognition of agency is required.

First, Farrer et al. (2008) observed that participants were more likely to report that visual feedback associated with an action had been modified when temporal contiguity was manipulated relative to when spatial contiguity was manipulated. Additionally, participants were far more unlikely to attribute the visual feedback to another person when temporal delays were applied relative to spatial deviation. These findings highlight that participants use multiple cues when making agency judgements and that, whilst participants may notice that their actions have been modified when temporal lags are applied to visual feedback, these are unlikely to influence attribution of an action to one’s self. Thus, manipulations of temporal contiguity may be better suited to investigating sensitivity to action modification rather than to the sense of agency. In contrast, paradigms manipulating spatial deviation and action-outcome contingency may be better suited to investigating the sense of agency.

With respect to action–outcome contingency, several experiments conducted mainly in the 1980s and early 1990s (e.g. Allan and Jenkins 1980, 1983; Alloy and Abramson, 1979; Chatlosh et al. 1985; Dickinson et al. 1984; Neunaber and Wasserman 1986; Shanks 1987, 1989; Shanks and Dickinson 1991; Shanks et al. 1989; Wasserman et al. 1983) demonstrated that action–outcome contingency was an important determinant of how much control participants considered they had over action outcomes. For example, Shanks and Dickinson (1991) tested several groups of participants, with each group able to cause an outcome to occur with a set probability (0.85) by pressing a button. The groups differed in the probability with which the outcome occurred in the absence of a button press, meaning that the probability of an outcome given an action was constant across groups, but the action–outcome contingency varied as a function of group. Results demonstrated that participants’ ratings of control (“did the button press result in the outcome?”) varied as a function of action–outcome contingency.

Despite their methodological sophistication, existing studies examining the effect of action–outcome contingency on the explicit sense of agency suffer from two main limitations. The first relates to trial numbers, which were extremely low by current standards (but see Allan and Jenkins 1983 for a notable exception). For example, participants in the Shanks and Dickinson (1991) study completed four judgement trials only (one at each level of contingency). Low trial numbers mean metacognition of agency is impossible to calculate in a reliable manner. The second relates to the nature of the paradigm employed. In the majority of these studies participants choose whether and how often they engage in an action across a given period, resulting in considerable individual differences in the proportion of time active. Indeed, participants engage in different strategies (e.g. some may act a lot to determine contingency whilst others may act very little to determine contingency), and these strategies differentially relate to their ability to accurately perceive action–outcome contingencies (Allan and Jenkins 1980, 1983; Wasserman et al. 1983). Therefore, low numbers of trials per participant and variability in action–outcome contingency information (due to differences in the nature of participant responses) mean that these studies are likely to provide an imprecise measure of the effect of action–outcome contingency on the explicit sense of agency at both the individual and group levels. Therefore, one of the aims of the current study was to confirm the impact of action–outcome contingency on the explicit sense of agency, and metacognition of agency, when participants complete more trials and action–outcome contingency information is kept constant across participants.

A second aim of the study was to differentiate the independent contribution of two other cues, spatial predictability and spatial deviation of action outcomes, to the sense of agency. Distinguishing the contribution of spatial predictability and deviation is important as previous work has often conflated the two. For example, studies show that the sense of agency can be reduced by introducing a spatial deviation between where the participant moves and the visual feedback associated with that movement (e.g. Ritterband-Rosenbaum et al. 2014). In such an experiment, it is not clear whether the spatial deviation or decreased predictability of action outcomes is responsible for the reduced sense of agency. In some paradigms, the sense of agency is compared when participants observe veridical visual feedback from their unseen actions, or false feedback from a previous movement (e.g. David et al. 2007; Yon et al. 2020). In these studies, it is not clear whether the reduced sense of agency felt while observing the false feedback is due to increased spatial deviation, reduced spatial predictability, or reductions in action–outcome contingency.

Spatial deviation has been shown to influence the sense of agency even when outcomes are predictable and always contingent on the agent’s actions (e.g. Fourneret and Jeannerod, 1998; Ritterband-Rosenbaum et al. 2014). However, the extent to which the spatial predictability of action outcomes influences the sense of agency (when controlling for spatial deviation and keeping contingency constant) remains untested. Thus, in the current study we also investigated the extent to which spatial predictability (when controlling for deviation) and spatial deviation (when controlling for predictability) influenced the explicit sense of agency. We predicted that both spatial deviation and action–outcome contingency would influence explicit agency judgements. No predictions were made for the influence of predictability on explicit agency judgements given the lack of prior evidence.

The final aim of this study was to investigate the influence of action–outcome contingency on metacognition of agency and the extent to which this varied in relation to various traits. Previous work has shown that temporal lags between an action and outcome, and spatial deviation of action outcome can both impact metacognition of agency (Metcalfe and Greene 2007). However, the extent to which action–outcome contingencies influence metacognition of agency remains untested (but see Shanks 1987, for early work on confidence judgements in high and low action–outcome contingency conditions). In our previous work, autistic traits were related to the degree that action–outcome contingency influenced motivation to act (Penton et al. 2018). Previous studies have also shown that schizotypy and attributional style relate to the sense of agency (Moore and Bravin 2015; Asai and Tanno 2007; Asai et al. 2008; Penton et al. 2014). Alexithymia has also been shown to correlate with the ability to monitor internal bodily signals (Murphy et al. 2018) and agency-related constructs such as locus of control (one’s tendency to attribute control to the self or external factors; Verissimo et al. 2000; Hexel, 2003). Further, studies have shown altered metacognition of agency in related conditions (e.g. autism—Zalla et al. 2015; psychosis—Krugwasser et al. 2022; schizophrenia—Metcalfe et al. 2012). Thus, understanding the relationship between these traits and metacognition of agency may help shed light on individual differences in the sense of agency and metacognition.

We therefore designed a new task in which we could manipulate action–outcome contingency, spatial predictability, and spatial deviation independently of each other (for other research investigating multiple cues to agency see Krugwasser et al. 2019; Farrer et al. 2008). We asked participants to rate their sense of agency, and their confidence in that rating, after each trial. Initial samples of participants were recruited to complete each of the three versions of the task (contingency, predictability, and deviation). A replication sample then completed shortened forms of all three versions of the task.

## Methods

### Sample sizes

All sample sizes in the current study are sufficient to detect medium to large effects with an alpha level of 0.05 and with 80% power. Effects of this size would be consistent with previous research investigating metacognition of agency (e.g. Metcalfe and Greene 2007 reported large effect sizes with a sample size of *N* = 24) and with each of the studies detailed in “Introduction” which assessed the impact of action–outcome contingency on the sense of agency. Sample size for the replication sample was sufficient to detect effects of an equivalent size to those seen for significant effects in the initial samples in our study with an alpha level of 0.05 and 80% power. Significant effects in the original samples in the current study were large (*d* ranging from 0.95–1.68) and, thus, sample sizes of *N* = 23 (contingency replication after outlier removal) and *N* = 26 (deviance and predictability replication after outlier removal) were sufficient to replicate these effects (the replication sample, after outlier removal, was sufficiently powered to detect medium–large effects where *d* > 0.62).

### General task methods

One task with three versions was used to assess the impact of contingency, predictability, and deviation on the sense of agency. Each version manipulated the relationship between the participant’s movements and the movement of an on-screen cursor. These manipulations are described in further detail below. The experimental task was created and presented using Matlab 8.0 (https://mathworks.com/) with the Cogent 2000 toolbox (http://www.vislab.ucl.ac.uk/Cogent).

**Fig. 1:**
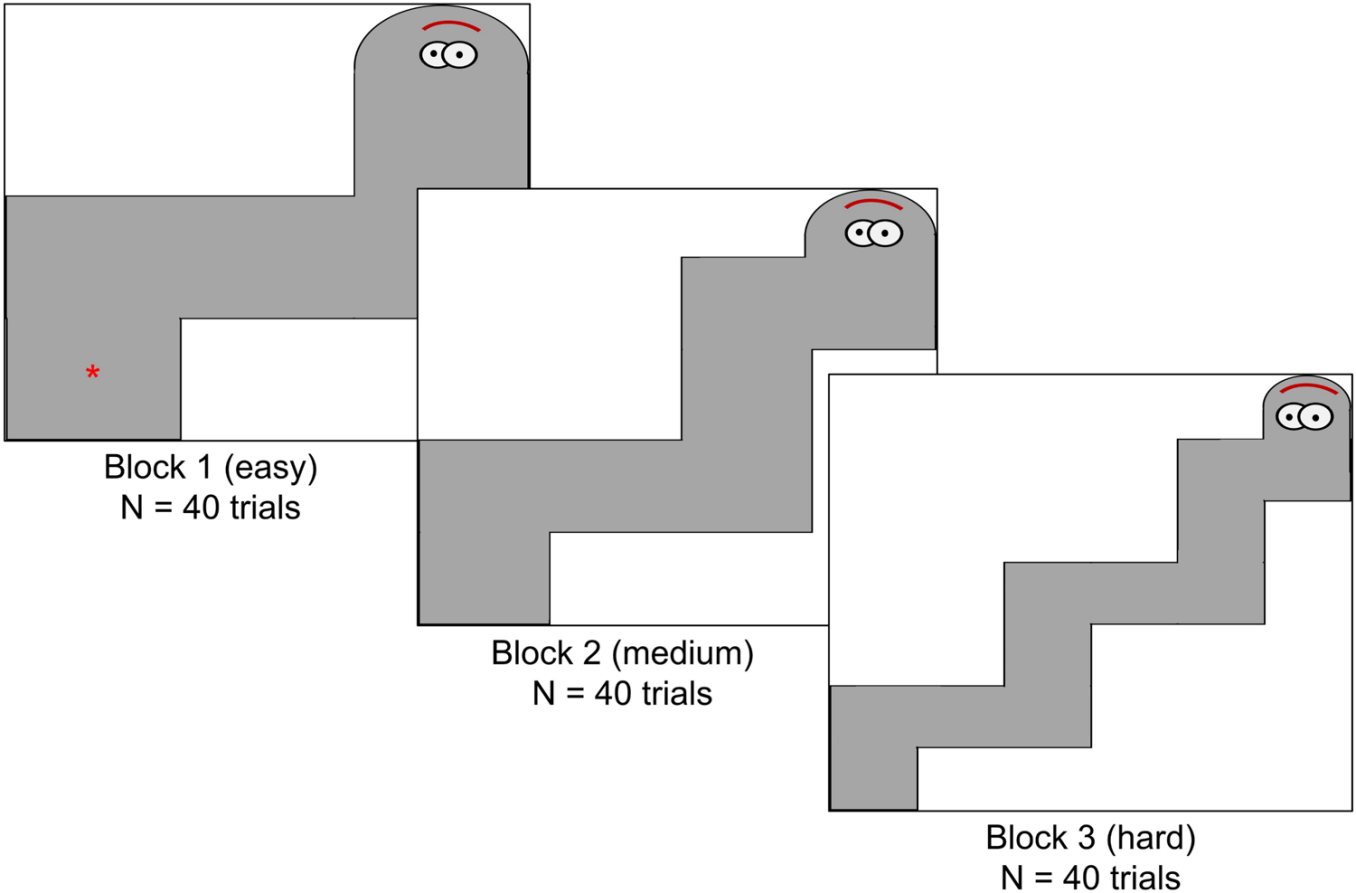
Typical snake stimulus presented on the screen for each of the three difficulty levels. The cursor (red star) can be seen in block one and is the relative size of the cursor participants saw during the trial

During all versions of the task, participants were asked to move a red cursor along a static snake on the screen using the trackpad on a laptop (see Fig. 1). The goal of each trial was to reach the top of the snake in the allotted time, taking care to keep the red cursor within the boundaries of the snake. In the Predictability and Deviance versions of the task, participants had 6 s to reach the top of the snake. In the contingency version, participants were allowed 12 s. The longer trial time for the contingency version was necessary as participants were required to remain still for 6 s of the trial due to the nature of the contingency manipulation (see below for further detail). Thus, whilst overall trial time differed between the contingency version and other versions of the task, the movement time was the same across all three versions of the task. Participants were asked to be as fast and as accurate as possible. Prior to beginning experimental trials, participants passively viewed six example trials. After each example, the participants were informed how the example trial was scored (% accuracy) and how this score was achieved. Accuracy was reduced if the cursor did not make it to the top of the snake, if the cursor moved outside the boundaries of the snake, and if the cursor moved when participants were instructed to stay still (contingency trials only—see below). The six example trials depicted a range of performances including high accuracy and low accuracy trials with different combinations of errors (i.e. the cursor made it to the top of the snake in the allotted time, but did not stay inside the boundaries of the snake; the cursor stayed inside the boundaries, but did not make it to the top of the snake). Participants then completed two practice trials without feedback as to their performance. At the end of each trial, participants were asked to indicate how accurate they were in the trial (0–100% accuracy) and how much control they had (no control—complete control) using a visual analogue scale. After each judgement, participants were also asked to indicate how certain they were of each judgement using a visual analogue scale (uncertain–certain). Order of the accuracy and control judgements was counterbalanced across participants and questions appeared immediately following each trial. Each version had three levels of difficulty, manipulated by adjusting the width of the snake (see Fig. 1). Participants completed all three levels (initial samples—see below) or just the most difficult level (replication samples—see below).

Participants were recruited from King’s College London using university-wide advertisements and were remunerated at a rate of £8 per hour. The study was approved by King’s College London Psychiatry, Nursing and Midwifery Research Ethics Subcommittee.

### Contingency version

Each trial lasted 12 s. In addition to moving the cursor to the top of the snake as fast and accurately as possible, participants were also asked to remain still in cases where the snake changed colour (from black to green). The trial time was equally split between green and black snake colours. Each colour had 10, 600 ms segments assigned to it and these were randomly dispersed throughout the trial. Cases where the participants were required to remain still and where participants were required to move were necessary to manipulate action–outcome contingency (i.e. the probability of the outcome occurring both when the participant was moving and when they were still). Specifically, 600 ms ‘freeze’ periods (cursor remains static when participant is moving when the snake is black) and 600 ms ‘jitter’ periods (cursor moves when the participant is still when the snake is green) were introduced randomly into trials. The amount of freeze and jitter periods in a given trial dictated the level of control the participant had over the cursor (1, 2, 3 or 4 possible freezes per trial; 1, 2, 3 or 4 possible jitters per trial, with the number of freezes always equal to the number of jitters). Four levels of contingency were utilised, corresponding to 1200, 2400, 3600, and 4800 ms of freeze/jitter. Thus, the movements of the cursor were not contingent on the participant’s movements for 10%, 20%, 30% or 40% of the total trial time.

#### Initial contingency sample

Forty-five participants completed the contingency version (see results for demographics). Participants completed 40 trials at each of the three difficulty levels (120 trials in total). This version of the task took approximately 1 h to complete. Participants were given the opportunity for a break at the end of each block of 40 trials.

### Predictability version

Each trial lasted 6 s. In this version, the number of possible locations that the cursor could appear was manipulated to influence how predictable the cursor position was. The possible locations in which the cursor could appear were always governed by the X and Y coordinate, which would be reached by the participant’s movement were it veridically represented by the cursor. The Y coordinate was always veridically represented (i.e. was not subject to manipulation). The position of the cursor in the X plane was governed by a matrix of positions of 60 pixels wide centred on the cursor’s veridical X coordinate. Where the cursor appeared within the matrix was determined randomly from a subset of positions within the matrix, where the number of possible positions varied according to the four levels of the predictability manipulation (5, 10, 30 or 60 possible locations randomly determined at the start of the trial). The cursor position was updated every 10 ms.

#### Initial predictability sample

Thirty-five participants completed the predictability version. Participants completed 40 trials at each of the three difficulty levels (120 trials in total). These participants also completed the deviation version (see below) in the same session (order counterbalanced). The session took approximately 1 h to complete (the predictability version took approximately 30 min). Due to a technical issue, data from two participants were not recorded, thus data from 33 participants were analysed. Participants were given the opportunity for a break at the end of each block of 40 trials.

### Deviation version

Each trial lasted 6 s. In this version, the degree of spatial deviation between the participant’s movement and the movement of the cursor was manipulated. Deviation was manipulated in the X plane only, and was achieved by calculating the difference between the cursor’s last position and where it would appear currently if the participant’s movement was veridically represented. This difference was multiplied by 2, 4, 6 or 8 to create the four levels of deviation manipulation, with the result used to determine the cursor’s position in the X plane. The cursor position was updated every 10 ms.

#### Initial deviation sample

Thirty-five participants completed the deviance version. Participants completed 40 trials in each of the three difficulty blocks (120 trials in total). Participants also completed the predictability version in the same session in a counterbalanced order. The deviation version took approximately 30 min to complete. Participants were given the opportunity for a break at the end of each block of 40 trials, and in between the two versions of the task (predictability or deviation—order counterbalanced).

### Replication sample

In a follow-up study, 28 new participants completed the third difficulty block only of each of the three versions of the task (see “Results” for demographics). The order of the three versions of the task was randomised across participants. Only the most difficult block was used to avoid ceiling effects on performance seen in some of the easier blocks in the initial samples. The contingency version of the task took approximately 30 min to complete whilst both the deviation and predictability versions of the task took 15 min to complete.

### Questionnaires

In addition to the computer-based tasks, participants who completed the contingency task also completed four questionnaires to assess individual differences in autistic traits (measured using the 50-item autism quotient AQ; Baron-Cohen et al. 2001), attributional style (measured using the attributional style questionnaire, ASQ; Peterson et al. 1982), schizotypy (measured using the Peters Delusion Inventory, PDI; Peters et al. 1995) and alexithymia (measured using the Toronto Alexithymia scale, TAS-20; Bagby et al. 1994). For a more detailed description of the measures see Penton et al. (2018).

### Analysis

Separate accuracy scores were calculated for the participant reaching the top of the snake (calculated as the percentage of the snake the cursor had moved through in the trial), for the cursor staying within the boundaries of the snake (the percentage of time during the trial that the cursor was within the boundaries) and, in the case of the contingency version, the cursor staying still during snake colour changes (calculated as the percentage of time the cursor was moving when it was required to be still). These percentages were averaged to create an overall percentage accuracy score for each trial. Control and certainty judgements were also expressed as percentages.

To calculate the effect of each of the three manipulations (contingency, predictability and deviation) a categorical value of 1–4 was assigned to each trial to indicate the degree of manipulation (1 = *lowest* degree of manipulation, 4 = *highest* degree of manipulation; hereafter ‘manipulation level’). Additionally, for the predictability version, the discrepancy between the veridical cursor position and where it was displayed was calculated along the *x* plane. This additional measure was calculated for the predictability version to allow for the removal of variance associated with deviation (i.e. so that the influence of predictability could be identified unconfounded by deviation).

#### Sense of agency (perceived control)

To investigate the relationship between each of the manipulated factors and perceived control, two regressions were conducted for each participant. Prior to calculating regression slopes, multivariate outliers were removed using Mahalanobis distance (calculating the multivariate distance between a point and the distribution; Mahalanobis 1930). For each participant, for each version of the task, an initial regression was conducted to calculate the degree to which perceived control was predicted by the manipulated variable. For the predictability manipulation, a regression was also conducted including deviation values to investigate the extent to which perceived control was predicted by predictability of cursor position after accounting for deviation. Following this initial regression, a second regression was conducted controlling for perceived performance. Perceived performance was included in the second regression model as judgements of performance are thought to impact one’s sense of agency (Metcalfe and Greene 2007). In all cases, negative betas reflect a greater sense of agency when control manipulations are smaller.

#### Metacognition and individual differences

The variable of interest in these analyses was metacognition for the sense of agency (i.e. the extent to which participants’ confidence in their judgements of control tracks the degree to which judgements of control were accurate). These values were calculated during the contingency version only, as this is the only version where control objectively varies, and therefore where the accuracy of control judgements can be calculated (during the predictability version the control of the cursor by the movement of the participant is fixed (the participant’s movement always determines the cursor’s Y coordinate and the centre of the matrix of fixed spatial extent), and during the deviation version the cursor’s location is controlled by the participant’s movements entirely). The individual differences variables were used to predict metacognitive values in separate regression models which either did, or did not, control for perceived performance.

The degree of actual control was calculated by determining the amount of time the participant was in control during a given trial. This was calculated by summing the durations of 600 ms freezes and jitters introduced to each trial and dividing this by the total trial time. This was then multiplied by 100 to get a percentage score representing the amount of time the participant was out of control. This value was then taken from 100 to get a percentage score reflecting the amount of time the participant was in control (i.e. an index of actual control).

To calculate metacognition of agency, the absolute difference between perceived control and actual control (percentage of time the participant was in control during the trial) was calculated. Higher scores indicate greater discrepancy between perceived and actual control and therefore indicate worse performance. These were then entered into a regression with certainty judgements to see the extent to which certainty judgements could predict accuracy of perceived control judgements. More negative betas indicate better metacognitive sensitivity for the sense of agency.

$$100 - \left( {\left( {\frac{{{\text{Time}}\,{\text{out}}\,{\text{of}}\,{\text{control}}}}{{{\text{Total}}\,{\text{trial}}\,{\text{time}}}}} \right) \times 100} \right)$$

## Results

### Task performance

#### Contingency

##### Initial sample

Forty-five participants completed all three difficulty blocks. Two participants were removed for outlying data (performing three times the interquartile range below the median). Thus, data for 43 participants were included in the analyses (mean age = 23.76, SD age = 6.4, 31 female). Mean accuracy for each block is presented in Table 1. A significant linear decrease in accuracy was observed as block difficulty increased [*F*(1,42) = 82.841, *p* < 0.001, *ƞp*^*2*^ = 0.664].

**Table 1:** Mean task accuracy (%) for all task versions for all blocks (initial samples) and for the replication samples

Task version	Initial samples			Replication
	Block 1	Block 2	Block 3	
Contingency	83.29% (2.22%)	82.58% (1.85%)	79.26% (2.74%)	75.90% (2.11%)
Predictability	96.73% (2.38%)	98.06% (1.39%)	96.16% (2.85%)	96.34% (1.87%)
Deviation	97.81% (1.94%)	98.39% (1.25%)	96.87% (1.97%)	95.08% (2.53%)

Standard deviation appears in parentheses

One-sample *t *tests were conducted at the group level to test whether beta coefficients were significantly different from 0. The contingency beta was significantly different from 0 in all three blocks (block 1 [*t*(42) = 10.109, *p* < 0.001, *d* = 1.54], block 2 [*t*(42) = 8.4, *p* < 0.001, *d* = 1.28], block 3 [*t*(42) = 9.65, *p* < 0.001, *d* = 1.47]). The contingency beta remained significantly different from 0 in all three blocks when controlling for perceived performance (block 1 [*t*(42) = 6.49, *p* < 0.001, *d* = 0.96], block 2 [*t*(42) = 5.04, *p* < 0.001, *d* = 0.77], block 3 [*t*(42) = 6.252, *p* < 0.001, *d* = 0.95]).

##### Contingency replication sample

Twenty-eight new participants completed the third difficulty block only. Data for five participants were not recorded due to a technical issue. Therefore, data for 23 participants were included in subsequent analyses (mean age = 28.47, SD age = 9.6, 18 female).

There was a significant difference in accuracy between the two groups [*t*(64) = 5.108, *p* < 0.001, *d* = 1.37]. This was due to a reduced accuracy in the replication sample relative to the initial sample [see Table 1]. This may reflect the fact that participants in the initial sample benefitted from ‘practising’ the task in the first two blocks.

As with the initial data, the contingency regressors were significantly different from 0, both when perceived performance was not controlled for [*t*(22) = 7.942, *p* < 0.001, *d* = 1.66] and when it was controlled for [*t*(22) = 5.424, *p* < 0.001, *d* = 1.13] [see Table 2]. No significant difference in beta weights between the two samples was found for either regression [(contingency regressor only: *t*(64) = 1.071, *p* = 0.288, *d* = 0.27), (contingency regressor controlling for perceived performance: *t*(64) = 1.711, *p* = 0.092, *d* = 0.43)]. This suggests that in both samples of participants, contingency affected the sense of agency to a similar extent.

**Table 2:** Average beta weights for all blocks (initial sample) and for the replication sample for the contingency version

	Initial sample			Replication
	Block 1	Block 2	Block 3	
Control (DV)/contingency (IV)	− 4.56 (2.96)*	− 4.93 (3.85)*	− 5.94 (4.03)*	− 7.08 (4.27)*
Control (DV)/contingency (IV) and performance (IV)	− 2.2 (2.23)*	− 2.18 (2.83)*	− 2.83 (2.97)*	− 4.28 (3.78)*

Standard deviation appears in parentheses

*Control* perceived control, *contingency* control manipulation, *performance* perceived performance, *DV* dependent variable, *IV* independent variable

Asterisks indicate betas significantly different from 0

#### Predictability

##### Predictability initial sample

Thirty-three participants completed the predictability version of the task. Two participants were removed for outlying accuracy data in the predictability version (performing three times the interquartile range below the median). Thus, data for thirty-one participants were included in the analysis (mean age = 22.33, SD age = 3.69, 26 female). The mean accuracy for each block is presented in Table 1. A significant decrease in accuracy was observed between the difficulty blocks, but this was reflective of a quadratic trend where participants were most accurate on the second difficulty block [*F*(1,30) = 36.163, *p* < 0.001, *ƞp*^*2*^ = 0.546] [see Table 1].

One-sample *t* tests were conducted to investigate whether predictability betas were significantly different from 0. Prior to controlling for deviation and perceived performance, the predictability beta was only significantly different from zero in the easiest block ([Block 1: *t*(30) = 3.568, p = 0.001, *d* = 0.64] but not in the more difficult blocks [Block 2: *t*(30) = 0.738, *p* = 0.466, *d* = 0.13; Block 3: *t*(30) = 0.682, p = 0.5, *d* = 0.12]. No betas from any of the difficulty blocks were significant after controlling for deviation and perceived performance [Block 1: *t*(30) = 0.303, p = 0.764, *d* = 0.05]; [Block 2: *t*(30) = 0.198, p = 0.844, *d* = 0.03]; [Block 3: *t*(30) = 0.294, p = 0.771, *d* = 0.05] [see Table 3].

**Table 3:** Average beta weights for all blocks (initial sample) and for the replication sample for the predictability version of the task

	Initial sample			Replication
	Block 1	Block 2	Block 3	
Control (DV)/predictability (IV)	− 1.54 (2.41)*	0.31 (2.32)	− 0.25(2.02)	0.66 (2.1)
Control (DV)/predictability (IV) and deviation (IV) and performance (IV)	− 0.35 (6.51)	− 0.25 (6.95)	− 0.32 (5.99)	3.41 (4.57)*

Standard deviation appears in parentheses

*Control* perceived control, *predictability* control manipulation, *deviation* deviation score, *performance* perceived performance, *DV* dependent variable, *IV* independent variable

Asterisks indicate betas significantly different from 0

##### Predictability replication sample

In a follow-up study 28 new participants completed the third difficulty block only. Due to a technical issue, data were not recorded for two participants, so 26 participants’ data were entered into subsequent analyses (mean age = 29.45, SD age = 10.31, 20 female). There was no significant difference in accuracy between the two samples [*t*(55) = 0.274, *p* = 0.785, *d* = 0.07] [see Table 1].

As with the initial data, the predictability beta (prior to controlling for deviation and performance) was not significantly different from zero [*t*(25) = 1.603, *p* = 0.121, *d* = 0.31]. However, in contrast to the initial data, the predictability beta was significantly different from zero after controlling for deviation and perceived performance [*t*(25) = 3.8, *p* = 0.001, *d* = 0.75]. However, in this case, the predictability beta was positive, indicating that participants perceive themselves as having more control as predictability was reduced (i.e. the opposite pattern of results to what would be expected if participants were using predictability as a cue to agency).

No significant sample difference in beta size was observed for the predictability beta prior to controlling for spatial deviation and perceived performance [*t*(55) = 1.658, *p* = 0.103, *d* = 0.44]; however, a significant difference between the two groups was found when controlling for deviation and perceived performance [*t*(55) = 2.597, *p* = 0.012, *d* = 0.7]. This reflects the fact that the second group had positive betas, while the first group had negative, but non-significant, betas.

#### Deviation

##### Deviation initial sample

Thirty-five participants completed the deviation version of the task. Five participants were removed due to outlying accuracy data (performing three times the interquartile range below the median). Two of these participants had trials where they did not move at all, so this poor performance was thought to indicate poor quality data rather than individual variability in the task. Thus, 30 participants’ data were included in the analysis (mean age = 22.38 years, SD age = 3.55, 24 female). Mean accuracy for each block is presented in Table 1. A significant linear trend on performance as difficulty increased was observed, reflective of participants performing worst on the most difficult block [*F*(1,29) = 4.431, *p* = 0.044, *ƞp*^*2*^ = 0.133] [see Table 1].

One-sample *t *tests were conducted to investigate whether betas were significantly different from 0 at the group level. The deviation manipulation beta was significantly different from 0 in all blocks {block 1 [t(29) = 10.303, p < 0.001, *d* = 1.88], block 2 [t(29) = 8.869, *p* < 0.001, *d* = 1.48], block 3 [t(29) = 9.174, *p* < 0.001, *d* = 1.68]}. The deviation manipulation beta remained significantly different from 0 in all blocks after controlling for perceived performance {block 1 [t(29) = 7.999, *p* < 0.001, *d* = 1.46], block 2 [t(29) = 6.938, p < 0.001, *d* = 1.27], block 3 [t(29) = 7.210, *p* < 0.001, *d* = 1.32]}.

##### Deviation replication sample

Twenty-eight new participants completed the third difficulty block only. Due to a technical issue, data from two participants did not record. Thus, data for 26 participants were included in the analysis (mean age = 29.45 years, SD = 10.31 years, 20 female). No outliers were removed from this analysis. There was a significant difference in accuracy between the two samples [*t*(54) = 2.968, *p* = 0.004, *d* = 0.79] [see Table 1]. As with the contingency task, this may be due to those in the initial group having benefited from ‘practising’ in the two prior blocks.

As with the initial sample, the deviation regressor was significantly different from zero [*t*(25) = 11.715, *p* < 0.001, *d* = 2.3], and remained significant when controlling for perceived performance [*t*(25) = 9.232, *p* < 0.001, *d* = 1.81].

Additionally, no significant difference in deviation manipulation beta between the two samples was found prior to controlling for performance [*t*(54) = 1.556, *p* = 0.126, *d* = 0.42]. However, a significant group difference in beta was observed after controlling for perceived performance [*t*(54) = 2.380, *p* = 0.021, *d* = 0.64]. This was due to a stronger effect in the replication sample relative to the initial sample (see Table 4).

**Table 4:** Average beta weights for all blocks (initial sample) and for the replication sample for the deviation version

	Initial sample			Replication
	Block 1	Block 2	Block 3	
Control (DV)/deviation (IV)	− 8.09 (4.3)*	− 8.8 (5.44)*	− 10.27 (6.13)*	− 12.71 (5.53)*
Control (DV)/deviation (IV) and performance (IV)	− 5.98 (4.1)*	− 5.68 (4.48)*	− 5.23 (3.97)*	− 7.88 (4.35)*

Standard deviation appears in parentheses

*Control* perceived control, *deviation* control manipulation, *performance* perceived performance, *DV* dependent variable, *IV* independent variable

Asterisks indicate betas significantly different from 0

### Metacognition and individual differences analyses

Data were pooled from the third block of the initial sample and the replication sample (*N* = 68) of the contingency version of the task. One participant was removed from the analysis for outlying data (performing more than three times the interquartile range below the median for the metacognition of agency analysis). Therefore, 67 participants were included in this analysis (mean age = 25.3 years, SD age = 7.79 years, 50 female).

As expected, negative betas were observed for the metacognition of agency regression (*M* = − 0.1, SD = 0.36). At the group level, the beta was significantly different from zero for metacognition of agency [*t*(66) = 2.196, *p* = 0.032, *d* = 2.67]. A positive beta was observed for the relationship between perceived control and perceived performance (*M* = 0.56, SD = 0.25). The relationship between perceived control and perceived performance was also significantly different from zero [*t*(66) = 18.293, *p* =  < 0.001, *d* = 2.24].

#### Individual difference analysis

The metacognition scores were entered into regressions with 4 individual differences measures (TAS-20, PDI, ASQ and AQ). One outlier was removed (as above). Additionally, not all participants completed all of the questionnaires. Sixty-one participants were included in the analyses investigating the ASQ and AQ (mean age = 25.3 years, SD age = 7.79 years, 47 female). Sixty participants were included in the analysis investigating the TAS-20 (mean age = 25.38 years, SD age = 7.83 years, 46 female), and 60 were included in the analysis investigating the PDI (mean age = 25.33 years, SD age = 7.85 years, 46 female). Note, one participant who completed the TAS-20 did not complete the PDI and vice versa. [See Table 5 for average questionnaire scores].

**Table 5:** Mean, standard deviation (in parentheses) and range of scores for the four individual differences questionnaires

	Mean (SD)	Range
TAS-20 (alexithymia)	47.45 (13.07)	23–83
PDI (schizotypy)	92.95 (56.84)	0–249
ASQ (attributional style)	0.27 (1.11)	− 2.67–2.39
AQ (autistic traits)	18.62 (8.26)	5–36

##### Metacognition of agency

Multiple linear regressions were conducted to investigate whether metacognition of agency significantly predicted response on the individual difference measures.^1^ The beta representing the relationship between perceived control and perceived performance was included to examine the unique variance explained by metacognition of agency. Additionally, average certainty judgements for the agency trials were included to control for the influence of metacognitive bias. All regressions are reported prior to, and following, controlling for additional variables. The assumption of multicollinearity was not violated for any of the four regressions for any of the predictors.

**Fig. 2:**
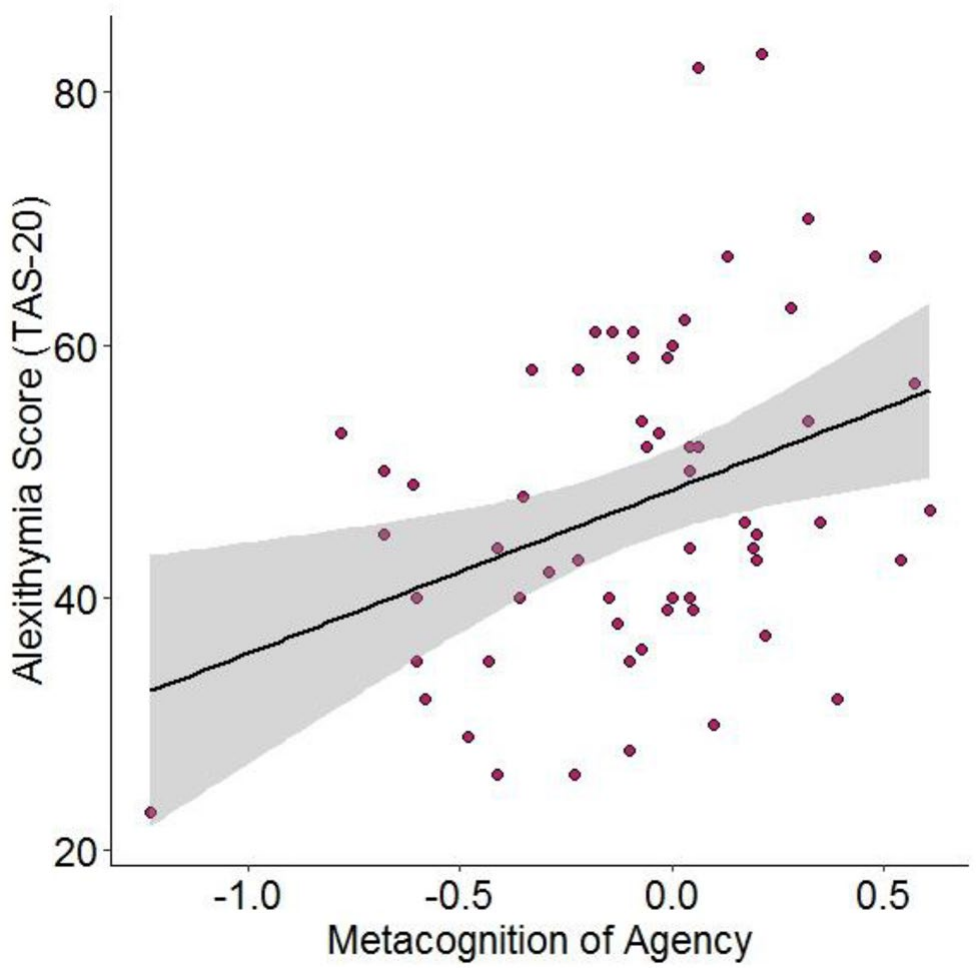
Relationship between metacognitive sensitivity for agency and alexithymia scores. Line represents line of best fit and shaded grey represents 95% confidence interval. No other individual difference scores were significantly predicted by metacognitive sensitivity for agency (see Supplemental Materials)

Metacognition of agency significantly predicted TAS-20 score (*b* = 0.355, *t* = 2.892, *p* = 0.005). Specifically, participants with poorer metacognitive sensitivity had higher alexithymia scores (see Fig. 2). Metacognition of agency remained a significant predictor of alexithymia score when controlling for the relationship between perceived control and perceived performance and average certainty judgements (bias towards being over or under confident on agency trials), *b* = 0.355, *t* = 2.629, *p* = 0.011. Neither the relationship between perceived control and perceived performance nor certainty judgements were significant predictors of TAS-20 score (perceived control and performance: *b* = 0.006, *t* =  − 0.047, *p* = 0.963; certainty: *b* = − 0.011, *t* = 0.084, *p* = 0.933).

## Discussion

In this paper, the sense of agency was measured when manipulating the relationship between action and outcome in three different ways. Results indicated that manipulations of contingency and spatial deviation of action outcomes, but not manipulations of the predictability of action outcomes, significantly impacted the sense of agency. For contingency, the relationship between the accuracy of participants’ sense of agency and their certainty in this judgement (metacognition of agency) was significant at the group level, and was significantly related to alexithymia, even when controlling for the relationship between perceived control and perceived performance, and average certainty judgements.

The results for the contingency version of the task suggest that participants’ sense of agency is sensitive to manipulations where both the probability of an outcome following an action and the probability of an outcome in the absence of an action are manipulated. Participants were also more certain when making more accurate control judgements, indicative of good metacognition of agency in the contingency version. Thus, these findings are consistent with previous research showing that participants are sensitive to changes in action-outcome contingency (e.g. Allan and Jenkins 1980, 1983; Alloy and Abramson 1979; Chatlosh et al. 1985; Dickinson et al. 1984; Neunaber and Wasserman, 1986; Shanks, 1987, 1989; Shanks and Dickinson 1991; Shanks et al. 1989; Sidarus et al. 2013; Wasserman et al. 1983).

Metacognition of agency for the contingency version of the task was a significant predictor of alexithymia score, even after controlling for the relationship between perceived control and performance and overall certainty bias. Specifically, worse metacognition of agency was associated with higher alexithymia scores. This is consistent with work showing a relationship between alexithymia and agency-related constructs such as internal locus of control and interoception (Murphy et al. 2018; Verissimo et al. 2000), and may point to a general failure to (meta) represent self-related information in alexithymia. Future research should therefore investigate the relationship between alexithymia and metacognition for other self-related judgements.

This task may be useful in understanding how cues related to action–outcome contingency influence the sense of agency in a range of clinical conditions. Studies in clinical groups often use spatial distortions, or temporal delays in paradigms investigating explicit sense of agency (e.g. psychosis—Krugwasser et al. 2022; schizophrenia—Metcalfe et al. 2012; Maeda et al. 2012; Parkinson’s disease—Saito et al. 2017). Whilst these studies give insight into how sensitive a person in a given clinical group is to perturbations to their own movement, they do not inform us about how different clinical groups interpret their control over an outcome *relative* to an external cause. Thus, the Contingency version of the snake task may have utility to further our understanding of the sense of agency in clinical groups. Future research should investigate whether this version of the task is sensitive to detecting disturbances in explicit sense of agency in clinical groups.

During the deviation version of the task, participants reported less of a sense of agency when the spatial deviation between the veridical and actual outcome of their action increased. This is in line with previous research showing that deviation between where the participant moves and the visual feedback associated with that movement impacts the sense of agency (Ritterband-Rosenbaum et al. 2014). In contrast to manipulations of contingency and spatial deviation, participants’ sense of agency was not impacted by the spatial predictability manipulation (in the initial sample) and even showed the opposite pattern of results to that expected in the replication sample (i.e. less spatially predictable outcomes were associated with greater perceived control). Importantly, this was not due to a general lack of attention, as control analyses showed that better performance was rated as more accurate [initial group: perceived and actual performance beta—*M* = 0.02, SD = 0.017; *t*(31) = 6.583, *p* < 0.001, *d* = 4.71; replication group: perceived and actual performance beta—*M* = 1.85, SD = 1.15; *t*(25) = 8.2, *p* < 0.001, *d* = 1.61]. This suggests that predictability, when controlling for deviation between intended and actual target location, may not be used when determining the sense of agency. In fact, in the case of the replication sample, spatial predictability may impair one’s ability to determine one’s sense of agency over the cursor. This highlights the need to account for the differential contribution of cues to agency—specifically to disambiguate spatial deviation and predictability.

## Conclusions

In conclusion, we demonstrate that the explicit sense of agency over the outcome of an action is affected by action–outcome contingency, and by the spatial deviation of the outcome, in that higher action–outcome contingencies and smaller spatial deviations were associated with a greater sense of agency. However, spatial predictability did not influence the sense of agency in the initial sample, and even appeared to impede agency judgements in the replication sample in which less spatial predictability was associated with a greater sense of agency. Furthermore, individual differences in alexithymic traits were related to metacognition of agency, suggesting that such judgements rely on the ability to monitor one’s internally generated signals associated with the performance of an action.

## Supplementary Information

Below is the link to the electronic supplementary material.

Supplementary file1 (DOCX 13 KB)

## Data Availability

The datasets generated and analysed during the current study are available in the OSF repository, https://osf.io/4ahyt/.

## Footnotes

1 Note: This regression allows the relationship between metacognition of agency and traits to be investigated whilst allowing for control variables to be added.
