# Supplementary material for: Investigating the sense of agency and its relation to subclinical traits using a novel task
Source: Exp Brain Res. 2022 Apr 5;240(5):1399–410. doi: 10.1007/s00221-022-06339-1 (PMC9038858; doi:10.1007/s00221-022-06339-1)
Supplement: Supplementary file 1 — Supplementary file1 (DOCX 13 KB) [file 221_2022_6339_MOESM1_ESM.docx]

**Supplemental Materials: Investigating the Sense of Agency and its Relation to Subclinical Traits Using a Novel Task**

The extent to which metacognition of agency predicted individual differences in schizotypy, attributional style, and autistic traits was explored. No individual difference scores were significantly predicted by metacognition of agency.

**Schizotypy**

Metacognition of agency did not significantly predict PDI scores (*b* = .098, *t* = 0.748, *p* = .458). Metacognition of agency remained a non-significant predictor when adding the relationship between perceived control and perceived performance and certainty judgements to the model (*b* = .06, *t* = 0.413, *p* = .681). Neither the relationship between perceived control and performance (*b* = .038, *t* = .265, *p* = .792) nor certainty judgements were significant predictors (*b* = .137, *t* = 1.020, *p* = .312).

**Attributional style**

Metacognition of agency did not significantly predict ASQ scores (*b* = -.015, *t* = 0.118, *p* = .906). Metacognition of agency remained a non-significant predictor when adding the relationship between perceived control and perceived performance and certainty judgements to the models (*b* = .009, *t* = 0.063, *p* = .950). Neither the relationship between perceived control and performance (*b* = -.023, *t* = 0.165, *p* = .869) nor certainty judgements were significant predictors (*b* = -.087, *t* = 0.651, *p* = .518).

**Autistic traits**

Metacognition of agency did not significantly predict AQ scores (*b* = -.014, *t* = 0.109, *p* = .914). Metacognition of agency remained a non-significant predictor when adding the relationship between perceived control and perceived performance and certainty judgements to the models (*b* = .052, *t* = 0.364, *p* = .718). Neither the relationship between perceived control and performance (*b* = .075, *t* = .532, *p* = .597) nor certainty judgements were significant predictors (*b* = .059, *t* = 0.440, *p* = .662).
